# Intravenous Transplantation of Mesenchymal Stem Cells Reduces the Number of Infiltrated Ly6C^+^ Cells but Enhances the Proportions Positive for BDNF, TNF-1*α*, and IL-1*β* in the Infarct Cortices of dMCAO Rats

**DOI:** 10.1155/2018/9207678

**Published:** 2018-10-02

**Authors:** Yunqian Guan, Xiaobo Li, Wenxiu Yu, Zhaohui Liang, Min Huang, Renchao Zhao, Chunsong Zhao, Yao Liu, Haiqiang Zou, Yanli Hao, Zhiguo Chen

**Affiliations:** ^1^Cell Therapy Center, Xuanwu Hospital, Capital Medical University, and Key Laboratory of Neurodegeneration, Ministry of Education, Beijing, China; ^2^Department of Neurology, Northern Jiangsu People's Hospital, Clinical Medical School of Yangzhou University, Yangzhou, China; ^3^Department of Neurology, The General Hospital of Guangzhou Military Command, Guangzhou, China; ^4^Department of Anatomy, Guangzhou Medical University, Guangzhou, China

## Abstract

The resident microglial and infiltrating cells from peripheral circulation are involved in the pathological processes of ischemia stroke and may be regulated by mesenchymal stem/stromal cell (MSC) transplantation. The present study is aimed at differentiating the neurotrophic and inflammatory roles played by microglial vs. infiltrating circulation-derived cells in the acute phase in rat ischemic brains and explore the influences of intravenously infused allogeneic MSCs. The ischemic brain injury was induced by distal middle cerebral artery occlusion (dMCAO) in SD rats, with or without MSC infusion in the same day following dMCAO. Circulation-derived infiltrating cells in the brain were identified by Ly6C, a majority of which were monocytes/macrophages. Without MSC transplantation, among the infiltrated Ly6C^+^ cells, some were positive for BDNF, IL-1*β*, or TNF-*α*. Following MSC infusion, the overall number of Ly6C^+^ infiltrated cells was reduced by 50%. In contrast, the proportions of infiltrated Ly6C^+^ cells coexpressing BDNF, IL-1*β*, or TNF-*α* were significantly enhanced. Interestingly, Ly6C^+^ cells in the infarct area could produce either neurotrophic factor BDNF or inflammatory cytokines (IL-1*β* or TNF-*α*), but not both. This suggests that the Ly6C^+^ cells may constitute heterogeneous populations which react differentially to the microenvironments in the infarct area. The changes in cellular composition in the infarct area may have contributed to the beneficial effect of MSC transplantation.

## 1. Introduction

Mesenchymal stem/stromal cells (MSCs) have a potential for treatment of neurological diseases, such as stroke. Bone marrow-derived MSCs (BM-MSCs) show merits in that they can be allogeneically or autologously transplanted without raising ethical or immunological problems.

The therapeutic mechanisms of intravenous MSC transplantation have been mainly ascribed to the neurotrophic and anti-inflammatory effects [1]. MSCs are capable of secreting a lot of cytokines and eliciting the host to produce many kinds of neurotrophic factors, including brain-derived neurotrophic factor (BDNF) [2], glial cell-derived neurotrophic factor (GDNF) [3], and insulin growth factor-1 (IGF-1) [4].

Inflammatory responses in stroke, including immune cells, cytokines, and chemokines, are important for stroke development and recovery. Local microglial cells and periphery circulation-derived cells, such as monocytes/macrophages, are important participants in stroke-induced inflammation and can be both beneficial and detrimental after stroke injury. Despite extensive studies on microglia in stroke, the roles of periphery-derived cells during ischemic stroke have not been fully characterized [5, 6].

We have previously shown a rapid accumulation of ionized calcium binding adaptor molecule-1- (Iba-1-) positive microglia in the injured cerebral cortex two days after dMCAO in rats [7]. After intravenous MSC transplantation, the concentrations of neurotrophin BDNF and proinflammatory cytokines TNF-*α* and IL-1*β* are all increased [7]. It will be interesting to examine the respective contribution from microglial vs. infiltrated cells in production of BDNF, TNF-*α*, and IL-1*β*.

However, microglial and infiltrating monocytes/macrophages share many features. Iba-1 expression does not allow complete discrimination of resident microglia from infiltrated monocytes/macrophages. Other commonly used pan-markers for microglia, such as CD11b, isolectin (IB4), and F4/80, are not specific enough either [6]. CD45 alone is not sufficient because although infiltrated hematopoietic cells express high levels of CD45, activated microglia express low levels of CD45 too [8].

It is therefore important to find a marker that can differentiate microglia from infiltrated monocytes/macrophages in rats. Ly6C^+^ cells represent circulation-derived monocytes/macrophages in mice and human [9, 10]. Whether the same holds true in rats is one of the questions that will be addressed in the current study.

One of the main therapeutic effects of MSCs after stroke is the neurotrophic effects [11]. Neurotrophins, such as BDNF, may be derived from the MSCs that have migrated into the brain, the endogenous neurons, and/or microglia/macrophages. BDNF is distributed in substantial amount in the ischemic core, peri-ischemic core cortex, ipsilateral striatum, and contralateral hemisphere [2, 12]. BDNF plays a crucial role in neuronal survival and plasticity, and its importance for stroke recovery has been demonstrated in Kurozumi's study. MSCs are transfected with BDNF, ciliary neurotrophic factor (CNTF), or neurotrophin 3 (NT3) vectors. Rats that undergo middle cerebral artery occlusion (MCAO) and receive MSC-BDNF exhibit significantly better functional recovery than do control stroke rats. MCAO rats that have received MSC-CNTF and MSC-NT3 do not show significant improvement vs. control stroke rats [13, 14].

We have previously found that in the ischemic brain, BDNF is expressed in NeuN-positive neurons and CD68-positive microglia/macrophages in the peri-infarct areas, but mainly by Iba-1-positive microglia in the infarct core [7]. Whether BDNF production can be derived from infiltrated cells and regulatable by MSC treatment is still unclear, and the current study will try to address these questions as well.

The other main therapeutic mechanism underlying MSC effect in stroke is the immunosuppressive effects, including downregulation of proinflammatory cytokines produced by microglia/macrophages [15]. Macrophage infiltration is normally thought to exacerbate focal inflammatory responses and further damage the brain by producing proinflammatory cytokines, such as TNF-*α* and IL-1*β* [16, 17]. As the most studied cytokines in adult stroke, IL-1*β* and TNF-*α* have been found to exacerbate brain damage by directly inducing neuronal injury and via consequent production of additional cytokines/chemokines and upregulation of adhesion molecules [18, 19]. Some groups found that IL-1*β* and TNF-*α* are expressed in largely segregated populations of CD11b^+^CD45dim microglia and CD11b^+^CD45high macrophages in mice [20].

In the current study, we will investigate whether neurotrophic factor BDNF and proinflammatory factors IL-1*β* and TNF-*α* are produced by infiltrated cells and how the production is regulated by MSC treatment.

## 2. Materials and Methods

### 2.1. Distal Middle Cerebral Artery Occlusion (dMCAO) Model, Peripheral Macrophage Depletion, and Cell Transplantation

The performance of allogeneic bone marrow MSC culture, cell transplantation, dMCAO model establishment, and behavioral tests have been described in our previous study [7]. In brief, 1 × 10^6^ MSCs in 1 mL 0.9% saline were administered via intravenous injection one hour after ischemia. One mL of 0.9% saline was given to the ischemia vehicle group (*n* = 10 per group).

Intravenous administration of clodronate liposomes was widely used for depletion of the monocyte/macrophage population in blood circulation. Clodronate liposomes do not affect CNS-resident microglia because they cannot pass the blood-brain barrier (BBB). In this study, clodronate liposomes (Liposoma BV, Amsterdam, Netherlands) were intraperitoneally injected 1, 2, and 3 days before the dMCAO. The dose of clodronate liposomes was 50 mg/kg according to the manufacturer's instructions. PBS injection was used as a negative control [21, 22].

The SD rats used in this study were divided into three groups, “sham” controls (skull was opened but without arterial occlusion), “ischemia + vehicle” group (dMCAO models with saline injection), and “ischemia + MSC” group (dMCAO models with MSC infusion). Three time points, 3, 24, and 48 h post-ischemia, were chosen. Under each condition, 5–10 rats were included.

### 2.2. Immunohistochemistry

The rats were anesthetized and transcranially perfused with 0.9% saline, followed by cold 4% formaldehyde (PFA). The brains were removed, postfixed in 4% PFA for 24 h, and stored in 30% sucrose/PBS at 4°C. All brains were sectioned on vibrating microtome at 40 *μ*m thickness. Free-floating sections across the entire brains were collected.

Immunohistochemistry and cell counting were performed as previously described [7]. In brief, floating brain sections were incubated in 0.3% Triton-100 PBS for 30 min and blocked with 2% donkey serum in PBS for 30 min at room temperature (RT). Sections were incubated overnight with Ly6C primary antibody (1 : 500 dilution, Santa Cruz Biotechnology, CA, USA). In the following day, sections were rinsed 3 × 15 minutes in TBS and incubated with FITC- or Cy3-conjugated secondary antibodies (1 : 300; Immune-Jackson Inc., CA, USA) for 2 h at RT. The sections were reblocked by 2% donkey serum in PBS for 30 min, then incubated with anti-CD45 (1 : 200 dilution, eBioscience, CA, USA), anti-CD68 (1 : 500 dilution, Chemicon, Temecula, CA, USA), biotin-conjugated anti-BDNF (1 : 500 dilution, R&D Systems, Minneapolis, USA), biotin-conjugated anti-TNF-*α* (1 : 500 dilution, R&D Systems, Minneapolis, USA), or biotin-conjugated anti-IL-1*β* antibodies (1 : 500 dilution, NeoBioscience Technology Co., Ltd, Shanghai, China). Other primary antibodies used included rat anti-rat Ly6C primary antibody (1 : 500 dilution, Santa Cruz Biotechnology, CA, USA), mouse anti-rat neutrophil elastase (1 : 500 dilution, Santa Cruz Biotechnology, CA, USA), and mouse anti-rat CD3 (1 : 500 dilution, NeoBioscience Technology Co., Ltd, Shanghai, China).

After being washed by PBS for 3 times, secondary antibodies were applied for 2 hours, followed by DAPI treatment for 20 min. Control reactions for antibody specificity were performed by omission of the primary antibodies. After being mounted onto slides, the positive cells were counted using a TCS SP5 II confocal laser scanning microscope (Leica, Wetzlar, Germany) at 200x magnification. The confocal settings, such as gain and offset, were designed to ensure that all pixels of all the selected sections were within the photomultiplier detection range. The setting was maintained to ensure all images were collected with the same parameters.

### 2.3. Cell Counting

In our experiments, the distribution of Ly6C, Iba-1, and BDNF was not restricted within the infarct area. For analysis, we counted the cells only in the cortical infarct areas.

**Figure 1:**
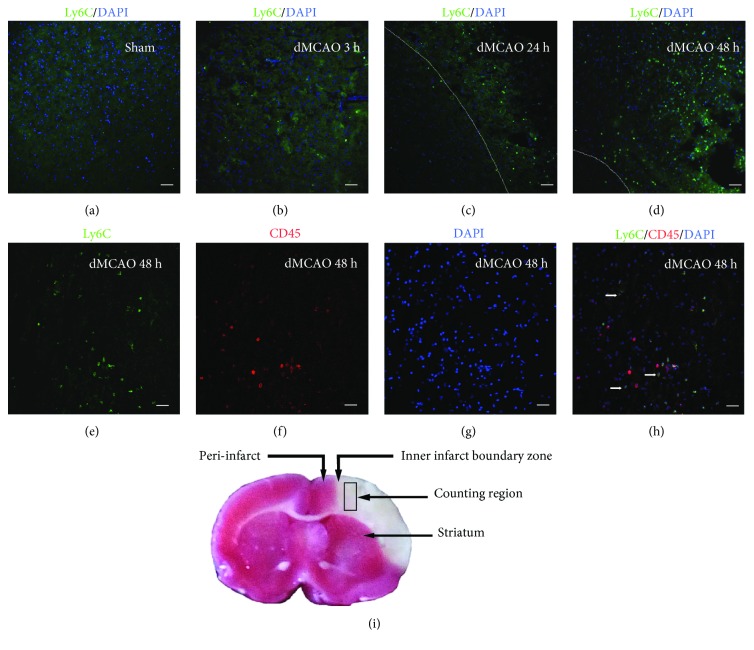
Ly6C^+^ cells in the ischemic core cortex. The number of Ly6C^+^ cells gradually increases at the IBZ of infarct dMCAO in rats. (a) Ly6C staining of the sham group. (b) Ly6C staining of the infarct area 3 h after the onset of ischemia. (c) 24 h. (d) 48 h. (e) Ly6C staining in the infarct area at 48 h. (f) CD45 staining in the same view field as (e). (g) DAPI nucleus staining. (h) Merged image of (e), (f), and (g), showing that Ly6C-positive cells are all CD45-positive. Arrow: double-labeled cells. (i) Cerebral infarct of the “ischemia + vehicle” group, as stained by triphenyltetrazolium chloride (TTC). Rectangle: the area where cell counting was performed. Dotted lines: the boundary of the infarct areas. Scale bar, 50 *μ*m.

The border zone between infarcted and healthy brain tissue is compartmentalized into an inner macrophage-rich part and a more peripheral zone dominated by reactive astrocytes [23, 24]. Based on this concept and the demarcation method of Gelosa et al. [25], we outlined the inner infarct boundary zone (IBZ) as within 400 *μ*m to the boundary line between the normal and infarct areas. The counting region is a 1.6 mm × 0.8 mm rectangle (rectangle in Figure 1(i)), which is located in the infarct cortex area and adjacent to the inner IBZ.

The selection of sections for each rat was in accordance with Simsek and Duman [26]. For each section, the Ly6C-positive and Ly6C/BDNF-, Ly6C/TNF-*α*-, and Ly6C/IL-1*β* double-positive cells that were located in the counting region were counted as previously described [26, 27]. The numbers of neurotrophils (neurotrophil elastase^+^) and T cells (CD3^+^) coexpressing BDNF, TNF-*α*, and IL-1*β* were also counted. The number of labeled cells was calculated in 3 coronal sections from each rat, located between −2.0 mm and 2.0 mm to the bregma and expressed as the mean number of cells per view field (20x objective). The estimated cell numbers were determined using the method of Simsek and Duman [26]. All analyses were performed by investigators blinded to the sample identity and treatment groups.

### 2.4. Statistics

The data of cell counting were expressed as mean ± SEM. The comparisons were analyzed by one-way analysis of variance (one-way ANOVA) and Bonferroni-Dunnett corrections using SPSS 10.0. The level of significance of all comparisons was set at *p* < 0.05.

## 3. Results

The success of ischemia model generation was validated by HE staining. The mortality rates of animals following dMCAO for the sham control, “ischemia + vehicle,” and “ischemia + MSCs” groups were 0, 16.7%, and 13.3%, respectively. There was no significant difference of mortality rates between the groups.

### 3.1. Ly6C^+^ Cells at the Infarct Area in Rat Brains Are Circulation-Derived Cells

Accumulation of Ly6C^+^ cells in the infarct area boundary zone (IBZ) was induced continuously over time following ischemia (Figures 1(a)–1(d)). The lineage identification is shown in Figures 1(e)–1(h), and the demarcation of inner IBZ and counting region was demonstrated in Figure 1(i).

Brain slices of naïve rats (no surgical operation, no dMCAO, and no MSC infusion) were stained, and no Ly6C^+^ cells in the cerebral cortex was detected, suggesting that the Ly6C^+^ cells observed in the dMCAO brains were derived from peripheral circulation (Figure S1A–D).

To further confirm the origin of Ly6C^+^ cells in dMCAO brains, we deleted the monocytes/macrophages in the periphery by i.p. injection of clodronate liposomes, which cannot cross BBB and therefore do not affect brain-resident microglia. Two days after dMCAO, the number of Ly6C^+^ cells in the cerebral cortex was reduced by 80–90% as compared with the sham group (Figure S1E–H).

In sham control rats, almost no Ly6C-expressing cells were found in the ischemia core cortex. Only after ischemia occurred did the Ly6C^+^ cells emerge in the infarct boundary zone (Figure 1(a)). Three hours after ischemia, Ly6C^+^ cells began to appear in the infarct area (Figure 1(b)). At 24 and 48 h, more and more Ly6C^+^ cells accumulated around almost the entire cerebral ischemia core cortex and striatum and were distributed widely in the ischemic hemisphere (Figures 1(c) and 1(d)). In contrast, few Ly6C^+^ cells were located in the ipsilateral cortex in the sham group 3, 24, and 48 h after ischemia.

At two days after the onset of ischemia, immunohistological examination showed strong expression of Ly6C in the infarct area (Figure 1(e)). The strong expression of CD45 (Figures 1(f)–1(h)) together with Ly6C reactivity confirmed that the cells were derived from periphery circulation.

### 3.2. Ly6C^+^ Cells Represent a Population Partly Overlapping with Iba-1^+^ Cells

To delineate the histological distribution and find out to what extent the two markers are overlapped, we performed double immunohistochemistry for Iba-1 and Ly6C.

**Figure 2:**
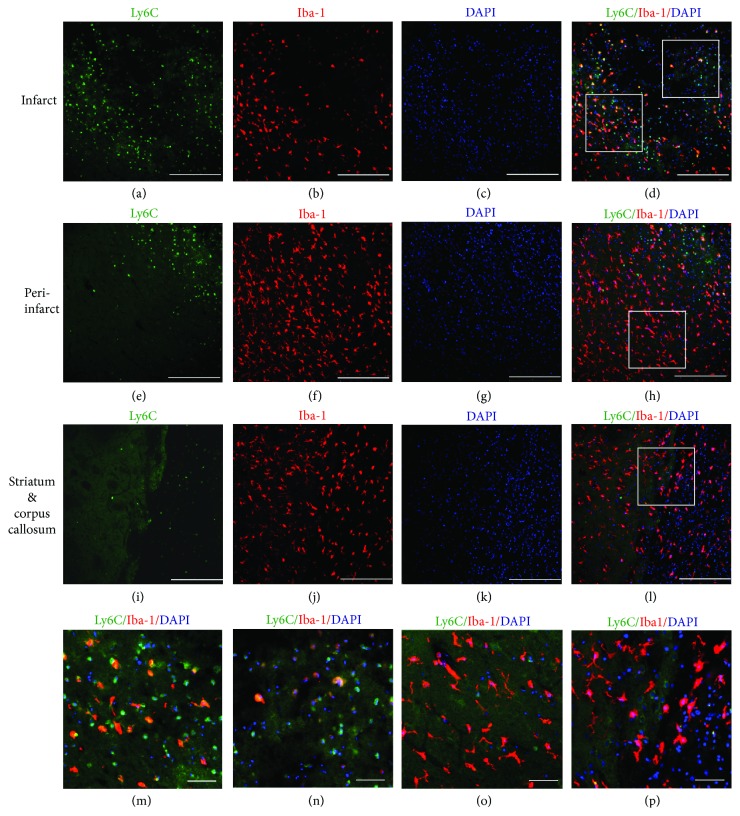
Few Ly6C^+^ cells are double-stained with Iba-1 in the ischemia core cortex at day 2. (a–d) Distribution of Ly6C^+^ and Iba-1^+^ cells in the infarct areas. (e–h) Distribution of Ly6C^+^ and Iba-1^+^ cells in the peri-infarct area of the ipsilateral cortex. (i–l) Distribution of Ly6C^+^ and Iba-1^+^ cells in the ipsilateral striatum and corpus callosum. (a, e, i) Ly6C staining. (b, f, j) Iba-1. (c, g, k) DAPI nucleus staining. (d, h, l) Double-stained Ly6C^+^/Iba-1^+^ cells. (d) Ly6C^+^/Iba-1^+^ cells scattered in the infarct area (right square) and the inner infarct boundary zone (left square). (h) Very few Ly6C^+^ cells were found in the ipsilateral peri-infarct area (to the left of the dashed line). (l) Very few Ly6C^+^ cells were found in the ipsilateral striatum (to the left of the dashed line) and corpus callosum (to the right of the dashed line), and no double stained Ly6C^+^/Iba-1^+^ cells were spotted in the striatum or corpus callosum. Arrow: double-labeled cells. Scale bar, 50 *μ*m. (m) (left square in (d)): in the inner infarct boundary zone, 30–40% of Ly6C^+^ cells were Iba-1-positive. (n) (right square in (d)): in the infarct area, 20–30% of Ly6C^+^ cells were Iba-1-positive. (o, p) (squares in (h) and (l)): very few or no double-stained Ly6C^+^/Iba-1^+^ cells was spotted in the striatum or corpus callosum. Scale bar: (a–l) 250 *μ*m and (m–p) 50 *μ*m.

A large portion of Ly6C^+^ cells were found accumulated in the inner infarct boundary zone and in the infarct area (Figures 2(a)–2(d)) after ischemia induction. The distribution of Iba-1^+^ cells was wider than that of Ly6C^+^ cells. Plenty of Iba-1^+^ cells were detected not only in the infarct area but also in the intact ipsilateral cortex (Figures 2(e)–2(h)), striatum, and corpus callosum (Figures 2(i)–2(l)) and even in the contralateral hemisphere. In the intact areas of both contra- and ipsilateral hemispheres, the majority of the Iba-1^+^ cells were of magnified morphology, and in the infarct, the predominant morphology of Iba-1^+^ cells was amoeboid-like round (Figures 2(d), 2(h), and 2(l)).

Only in the infarct cortex and the inner IBZ were a few Ly6C^+^ cells reactive for Iba-1 (Figures 2(a)–2(d)). The percentage of double labeled Iba-1^+^/Ly6C^+^ cells in the infarct area and the inner IBZ was 30.18 ± 8.57% and 30.91 ± 15.56% among the Iba-1^+^ cells, respectively (Figures 2(m) and 2(n)). Among the Ly6C^+^ cells, about 25.35 ± 3.26% coexpressed Iba-1 in the infarct area and 24.68 ± 4.14% coexpressed Iba-1 in the IBZ. There was no significant difference between the numbers at the infarct area and the inner IBZ. After MSC transplantation, the overlapping percentage of Iba-1^+^ and Ly6C^+^ cells was not changed in the infarct area and inner IBZ.

In peri-infarct area (Figures 2(e)–2(h)), striatum, and corpus callosum (Figures 2(i)–2(l)), few Ly6C^+^ cells were found, and they were negative for Iba-1 (Figures 2(o) and 2(p)).

When all of the Iba-1^+^ cells, including the contra- and ipsilateral cortices, striatum, and corpus callosum, were taken into account, the overlapped proportion with Ly6C was less than 0.01% among the Iba-1^+^ cells.

### 3.3. Ly6C^+^ Cell Is One of the Major Contributors for BDNF Production

We have shown that in the ipsilateral ischemia core cortex, Iba-1^+^ cells are one of the main sources for the production of BDNF [7]. Other cell types, such as CD68^+^ microglia, and NeuN^+^ neurons, are partly responsible for production of BDNF, even after the induction of ischemia. But all of these cells together do not represent the entire BDNF^+^ population. These results strongly suggest that there are other cell types as the source of BDNF in the infarct region.

**Figure 3:**
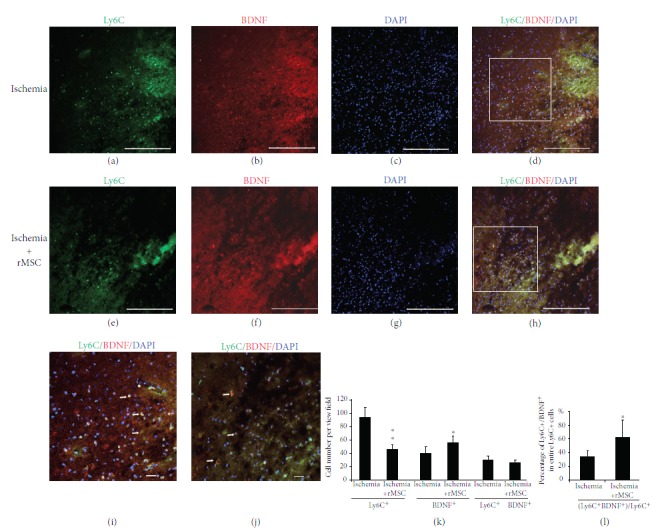
Ly6C^+^ cells are one of the major contributors for BDNF production at day 2. (a–d) Distribution of Ly6C^+^ and BDNF^+^ cells in the infarct before MSC transplantation. (e–h) Distribution of Ly6C^+^ and BDNF^+^ cells in the infarct after MSC transplantation. (a, e) Ly6C staining. (b, f) BDNF. (c, g) DAPI nucleus staining. (d) Double-stained Ly6C^+^/BDNF^+^ cells scattered in the infarct area after dMCAO. (h) Ly6C^+^/BDNF^+^ cells 2 days after MSC transplantation. (i, j) Squares in (d) and (h). Ly6C^+^ cell number was reduced in the infarct area 2 days after MSC transplantation, and the quantity and percentage of double-stained BDNF^+^ Ly6C^+^ cells were increased in these areas. (k) The cell counting results of Ly6C^+^, BDNF^+^, and Ly6C^+^/BDNF^+^ cells before and after MSC transplantation. (l) The percentage of Ly6C^+^/BDNF^+^ cells among Ly6C^+^ cells was increased by MSC treatment. (^∗^ *p* < 0.05 and ^∗∗^ *p* < 0.01, as compared with the ischemia vehicle group). Arrow: double-labeled cells. *n* = 5; scale bar: (a–h) 250 *μ*m and (i, j) 50 *μ*m.

We performed double immunohistochemistry for BDNF and Ly6C (Figure 3) and found that, two days after ischemia onset, BDNF expression was enhanced in infiltrating Ly6C^+^ cells in the ischemia core cortex as compared with the sham control group, and only in the infarct area was BDNF staining found overlapped with Ly6C (Figures 3(a)–3(d)). The numbers of Ly6C^+^ cells, BDNF single-positive cells, Ly6C^+^/BDNF^+^ double-positive cells were 93.8 ± 14.56, 40.2 ± 9.59, and 29.7 ± 6.18 per view field, respectively (Figure 3(k)).

MSC treatment reduced the quantity of Ly6C^+^ cells that had infiltrated into the brain to 35.7 ± 7.04 per view field, but increased the number of BDNF^+^ cells to 55.7 ± 10.10 (Figures 3(e)–3(k)). MSC transplantation did not change the number of Ly6C^+^/BDNF^+^ double-positive cells (25.5 + 4.45), but increased the proportion of Ly6C^+^ cells coexpressing BDNF from 32.40 ± 7.25% before MSC treatment to 71.65 ± 4.26% after treatment (Figures 3(i), 3(j), and 3(l)).

We have previously reported that BDNF expression was enhanced in Iba-1^+^ cells following ischemia [7]. Although around 25% of Ly6C^+^ cells were also Iba-1-positive in the infarct area, the current study strongly suggested that Ly6C^+^ cells may be another major contributor for BDNF production since the proportion of Ly6C^+^ cells coexpressing BDNF was dramatically enhanced to 71.65 ± 4.26% after MSC treatment.

### 3.4. Ly6C^+^ Cells Positive for IL-1*β* Are Upregulated by MSC Treatment

**Figure 4:**
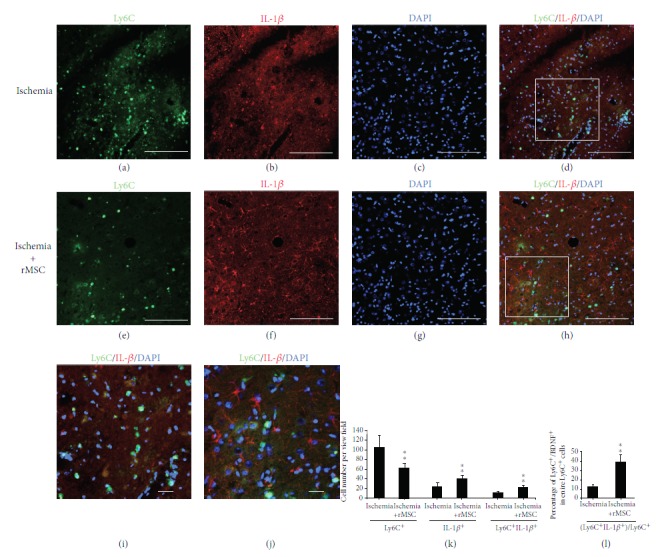
MSC treatment reduces the quantity of Ly6C^+^ cells but increases the proportion that produces IL-1*β* at day 2. (a–d) Distribution of Ly6C^+^ and IL-1*β* ^+^ cells in the infarct before MSC treatment. (e–h) Distribution of Ly6C^+^ and IL-1*β* ^+^ cells in the infarct after MSC treatment. (a, e) Ly6C staining. (b, f) IL-1*β* staining. (c, g) DAPI nucleus staining. (d) Double-stained Ly6C^+^/IL-1*β* ^+^ cells scattered in the infarct area 2 days after dMCAO. (h) Ly6C^+^/IL-1*β* ^+^ cells in the infarct area 2 days after MSC transplantation. (i) The magnified image of the square in (d). (j) The magnified image of the square in (h). (k) Ly6C^+^ cell number was reduced in the infarct area 2 days after MSC transplantation, but the quantity of IL-1*β* ^+^ cells and IL-1*β* ^+^/Ly6C^+^ cells was increased in the infarct area. (l) The percentage of Ly6C/IL-1*β* double-positive cells among the whole Ly6C^+^ cells was increased by MSC treatment. (^∗^ *p* < 0.05 and ^∗∗^ *p* < 0.01, as compared with the ischemia vehicle group). Arrow: double-labeled cells. *n* = 5; scale bar: (a–h) 250 *μ*m and (i, j) 50 *μ*m.

We examined IL-1*β* reactivity in Ly6C^+^ cells in the ischemia vehicle group (Figures 4(a)–4(d)) and in the ischemia + MSC group (Figures 4(e)–4(h)). The ischemia vehicle group showed a higher level of IL-1*β* compared to the sham control group (data not shown, *p* < 0.01), as evidenced by the increased number of IL-1*β* ^**+**^/Ly6C^+^ cells in the infarct area.

MSC transplantation reduced the number of Ly6C^+^ cells infiltrated into the brain but increased the number of IL-1*β* ^+^ cells from 24.75 ± 7.35 per view field before MSC transplantation to 41 ± 6.82 after transplantation (*p* < 0.01, Figure 4(k)). After all, the quantity of IL-1*β* ^+^/Ly6C^+^ double-positive cells was increased by MSC treatment from 12.5 ± 2.18 to 23.75 ± 3.16 per view field (*p* < 0.01, Figure 4(k)). The percentage of IL-1*β* ^+^/Ly6C^+^ double-positive cells in the entire Ly6C^+^ cells was also increased by MSC treatment from 12.30 ± 2.03% to 38.62 ± 7.99% (*p* < 0.01, Figure 4(l)).

We have previously reported that IL-1*β* protein level was enhanced in the infarct area 48 h after MSC transplantation [7]. The results from the current study suggest that Ly6C^+^ infiltrated cells in the brain may have contributed to this enhancement.

### 3.5. MSC Treatment Induces TNF-*α* Expression in Ly6C^+^ Cells

**Figure 5:**
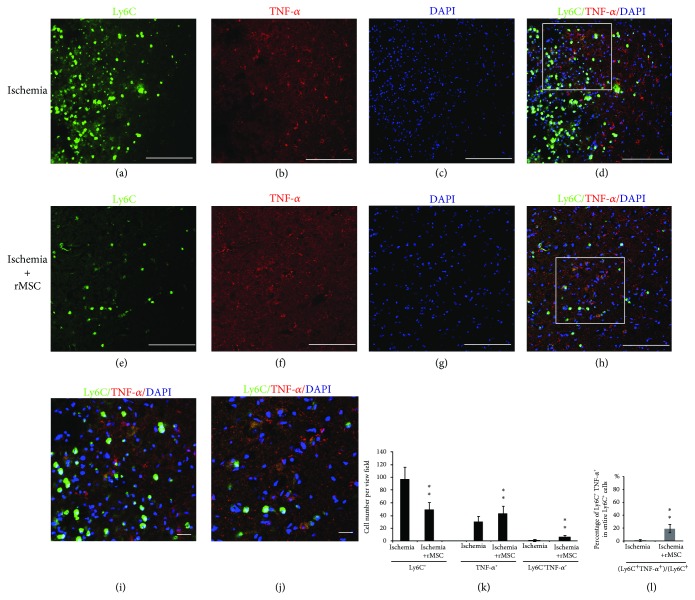
MSC increases the proportion of Ly6C^+^ cells coexpressing TNF-*α* in the ischemia core of the cortex at day 2. (a–d) Distribution of Ly6C^+^ and TNF-*α* ^+^ cells in the infarct area before MSC treatment. (e–h) Distribution of Ly6C^+^ and TNF-*α* ^+^ cells in the infarct area after MSC treatment. (a, e) Ly6C staining. (b, f) TNF-*α* staining. (c, g) DAPI nucleus staining. (d) Double-stained Ly6C^+^/TNF-*α* ^+^ cells scattered in the infarct area 2 days after dMCAO. (h) Ly6C^+^/TNF-*α* ^+^ cells in the infarct area 2 days after MSC transplantation. (i) The magnified image of the square in (d). (j) The magnified image of the square in (h). (k) The quantities of Ly6C^+^, TNF-*α* ^+^, and TNF-*α* ^+^/Ly6C^+^ double-positive cells were increased in the infarct area. (l) The percentage of TNF-*α* ^+^/Ly6C^+^ double-positive cells among the entire Ly6C^+^ cells was increased by MSC treatment. (^∗^ *p* < 0.05 and ^∗∗^ *p* < 0.01, as compared with the ischemia vehicle group). Arrow: double-labeled cells. *n* = 5; scale bar: (a–h) 250 *μ*m, (i, j) 50 *μ*m.

It was previously reported that in mouse stroke models, microglia are the main producer of TNF-*α* while macrophages are of IL-1(b) [20]. We examined TNF-*α* reactivity in Ly6C^+^ cells in the ischemia vehicle group and in the ischemia + MSC group. In the ischemia vehicle group (Figures 5(a)–5(d)), few TNF-*α* ^+^/Ly6C^+^ double-positive cells were detected in the infarct area (Figures 5(d) and 5(i)). The numbers of TNF-*α* single-positive and TNF-*α* ^+^/Ly6C^+^ double-positive cells were 31 ± 7.87 and 1.0 ± 1.73 per view field in the ischemia vehicle group, and the proportion of Ly6C^+^ cells coexpressing TNF-*α* was 0.9 ± 1.2% (Figures 5(k) and 5(l)).

After MSC treatment, TNF-*α* expression was upregulated (Figures 5(e)–5(h)). The distribution of TNF-*α* ^+^/Ly6C^+^ cells was still restricted within the infarct area and was not changed by MSC treatment (Figure 5(j)). The proportion of Ly6C^+^ cells coexpressing TNF-*α* was significantly increased (19.67 ± 6.54%) vs. the ischemia vehicle group (0.9 ± 2.2%, *p* < 0.01) (Figure 5(l)). Also, the number of TNF-*α* ^+^/Ly6C^+^ double stained cells was enhanced to 7.5±0.87 per view field (Figure 5(k)).

### 3.6. Iba-1^+^ Cells Are Not the Source of TNF-*α* or IL-1*β* Production in Rat Ischemia Brains and Cannot Be Induced by MSC Treatment

After the onset of ischemia in rats, in the contralateral hemisphere of the ischemia vehicle group, the Iba-1^+^ cells with resting morphology were found in the intact cortex, striatum, and corpus callosum, but in these areas, TNF-*α* or IL-*β* reactivity was not detected (image not shown). In the ipsilateral hemisphere, Iba-1^+^ microglia of both activated and resting forms were detected, but the resting phenotype existed in the infarct core cortex and intact striatum, whereas the active form was only found in the infarct core cortex.

**Figure 6:**
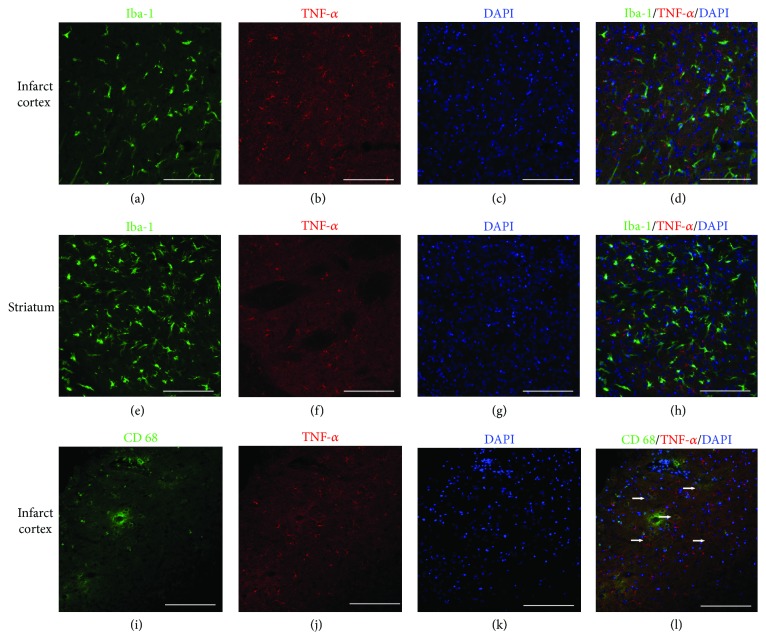
CD68^+^ microglia but not Iba-1^+^ cells express TNF-*α* in the ischemia ipsilateral hemisphere at day 2. (a–d) Distribution of Iba-1^+^ and TNF-*α* ^+^ cells in the infarct areas after MSC transplantation. (e–h) Distribution of Iba-1^+^ and TNF-*α* ^+^ cells in the striatum after MSC transplantation. (i–l) Distribution of CD68^+^ and TNF-*α* ^+^ cells in the infarct areas after MSC transplantation. (a, e) Iba-1 staining. (i) CD68 staining. (b, f, j) TNF-*α*. (c, g, k) DAPI nucleus staining. (d) No double-stained Iba-1^+^/TNF-*α* ^+^ cells were found in the infarct area after dMCAO. (h) No Iba-1+/TNF-*α* cells was found 2 days after MSC transplantation in the striatum. (l) Some CD68^+^ cells in the infarct areas were positive for TNF-*α*. Arrow: double-labeled cells. Scale bar, 250 *μ*m.

In the infarct area, the spatial distribution of Iba-1 signals was correlated with those of TNF-*α* (Figures 6(a)–6(h)), although the two signals were never overlapped, whether in the infarct area (Figures 6(a)–6(d)) or the intact striatum and corpus callosum (Figures 6(e)–6(h)). Some CD68^+^ cells in the infarct area (possibly activated microglia) were positive for TNF-*α* (Figures 6(i)–6(l)).

**Figure 7:**
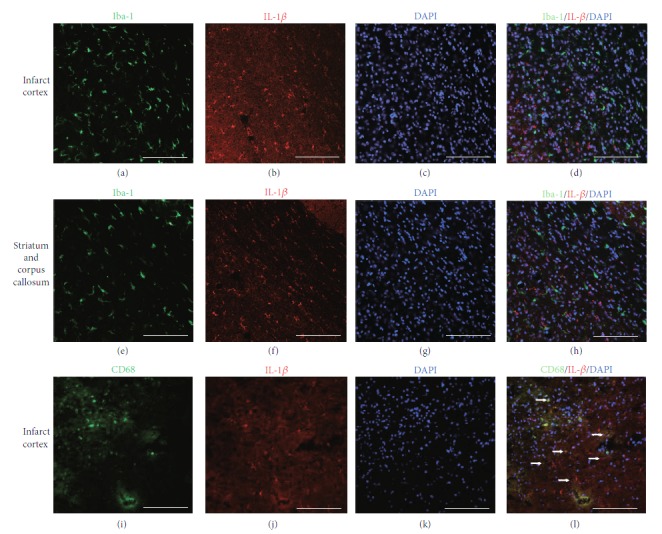
CD68^+^ microglia but not Iba-1^+^ cells express IL-*β* in the ischemic ipsilateral hemisphere at day 2. (a–d) Distribution of Iba-1^+^ cells and IL-*β* ^+^ cells in the infarct areas after MSC transplantation. (e–h) Distribution of Iba-1^+^ cells and IL-*β* ^+^ cells in the striatum after MSC transplantation. (i–l) Distribution of CD68^+^ cells and IL-*β* ^+^ cells in the infarct areas after MSC transplantation. (a, e) Iba-1 staining. (i) CD68 staining. (b, f, j) IL-*β*. (c, g, k) DAPI nucleus staining. (d) No double-stained Iba-1^+^/IL-*β* ^+^ cells were found in the infarct area after dMCAO. (h) No Iba-1^+^/IL-*β* ^+^ cells were found 2 days after MSC transplantation in the striatum and corpus callosum after MSC transplantation. (i–l) Some CD68^+^ cells were positive for IL-*β*. Arrow: double-labeled cells. Scale bar, 250 *μ*m.

Similarly, the distribution of Iba-1^+^ and IL-*β* ^+^ cells was spatially correlated but not overlapped (Figures 7(a)–7(h)). We have previously reported that the number of Iba-1^+^ cells can be regulated by MSC infusion [7]. The current results indicated that in mild damage such as dMCAO, MSC treatment did not alter production levels of TNF-*α* or IL-*β* from Iba-1^+^ cells. Iba-1^+^ cells might not be the target of MSC regulation with respect to secretion of proinflammatory cytokines TNF-*α* and IL-*β*.

### 3.7. BDNF-Positive Cells Do Not Overlap with TNF-*α*- or IL-1*β*-Positive Cells

Ischemia-induced accumulation of Ly6C^+^ cells was localized in the ischemia core cortex and the surrounding areas, the same region where BDNF^+^ cells resided. Here raises a question of whether BDNF and proinflammatory cytokines, such as TNF-*α* or IL-*β*, can be produced in the same cell positive for Ly6C.

**Figure 8:**
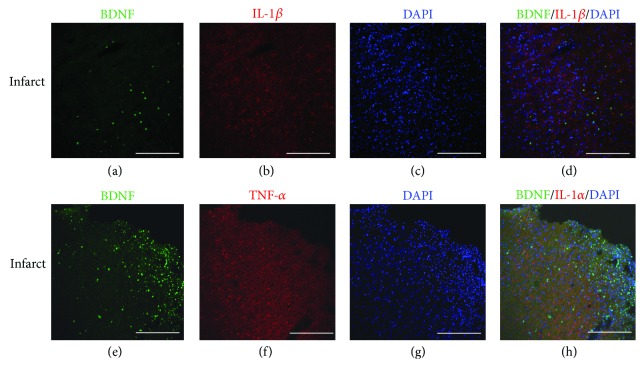
BDNF-producing cells do not overlap with TNF-*α*-or IL-1b-expressing cells at day 2. (a–d) Distribution of BDNF^+^ cells and IL-*β* ^+^ cells in the infarct areas after MSC transplantation. (e–h) Distribution of BDNF^+^ cells and TNF-*α* ^+^ cells in the infarct area after MSC transplantation. (a, e) BDNF staining. (b) IL-*β*. (f) TNF-*α*. (c, g) DAPI nucleus staining. (d) No double-stained BDNF^+^/IL-*β* ^+^ cells were found in the infarct area after dMCAO. (h) No BDNF^+^/TNF-*α* ^+^ cells were found 2 days after MSC transplantation in the striatum and corpus callosum after MSC transplantation. Scale bar, 250 *μ*m.

We examined BDNF reactivity in TNF-*α* ^+^ and IL-*β* ^+^ cells (not restricted to macrophages) two days after stroke. No BDNF^+^ cells were double labeled with TNF-*α* ^+^ or IL-*β* ^+^ cells in the brain, whether at the ischemia damaged areas or the intact areas (Figure 8).

### 3.8. Ly6C^+^ Cells Are Partly Positive for Neutrophil Antigen Neutrophil Elastase (NE) or T Cell Antigen CD3

We also tried to identify the cell populations that comprise the Ly6C^+^ cells in the ischemic brain. Since most of the granulocytes are neutrophils in the blood of rats, we selected neutrophil elastase (NE), to identify neutrophils in the brain. Approximately 8.62 ± 2.62% of Ly6C^+^ cells coexpressed NE in the infarct areas (Figure S2A–D). MSC transplantation did not change the proportion significantly (9.31 ± 2.45%).

We also examined the coexpression of Ly6C and T cell marker CD3. Around 15.21 ± 4.62% of Ly6C^+^ cells in the infarct areas coexpressed CD3 (Figure S2E–H). MSC transplantation did not significantly change the proportion either (17.31 ± 5.45%).

Here raised another question—could these overlapping cells also contribute to the production of BDNF, TNF-*α*, and IL-1*β* following dMCAO? We found that very few NE- or CD3-positive cells were double-stained with BDNF (<1%) (Figure S3A–H). In terms of IL-1*β*, it was expressed by nearly 2% NE^+^ neutrophils and 3–5% CD3^+^ T cells (Figure S4A–H). Only 1–2% NE^+^ neutrophils and 2–4% CD3^+^ T cells expressed TNF-*α* (Figure S5A–H).

The results suggest that it is probably the infiltrating monocytes/macrophages that are the major contributors for Ly6C^+^ cells that coexpress BDNF, TNF-*α*, or IL-1*β*.

## 4. Discussion

In the acute and subacute phases, resident microglial activation [28] and infiltration of circulating leukocytes to the ischemic brain [29] are the key features of the neuroimmunological responses to brain ischemia.

Previously, both microglia and infiltrated circulating leukocytes have been considered by many researchers to be harmful to the brain, since inhibition of some of these inflammatory responses improved the outcome in animal experiments. However, clinical trials aimed at inhibiting microglial activation or preventing leukocyte trafficking into the ischemic brain were unsuccessful [30]. It is nowadays accepted that the roles of inflammation in stroke are complicated in that they play both harmful and protective functions. This may be one reason why previous clinical trials of inhibiting immunological responses in stroke uniformly failed [30, 31].

Immune cells present in the healthy CNS and those recruited into stroke lesions are heterogeneous, including neutrophils, monocytes, macrophages, dendritic cells, T and B lymphocytes, and natural killer cells. Among these cells, monocytes/macrophages are important in the development of cerebral ischemia damages.

Given the importance of microglia and monocytes/macrophages in the stroke damage and that the marker “Iba-1” is normally expressed by both microglia and macrophages, it is important to identify a specific marker for infiltrated monocytes/macrophages in the ischemic brain.

Since Ly6C is widely used as the marker of mouse and human monocytes/macrophages, we selected Ly6C in this rat stroke study. Firstly, Ly6C^+^ cells were not detected in brains of naïve rats and were only detected in brains of dMCAO rats. Secondly, depletion of peripheral immune cells by treatment with clodronate liposomes led to 80–90% reduction in the number of Ly6C^+^ cells in dMCAO brains. Together with the observation that Ly6C^+^ cells were also CD45^+^ in infarcted rat brains, it is suggested that Ly6C^+^ cells in the brain represent circulation-derived infiltrated cells.

In ischemic brains, around 25% of Ly6C^+^ cells were double-positive for Iba-1, and these double-stained cells were only detected inside the infarct areas. It is possible that some of the infiltrating Ly6C^+^ immune cells may become Iba-1^+^ over time.

CD68 is another important microglial marker. In our study, although, functionally, CD68^+^ cells were a separated population from Iba-1^+^ cells, morphologically, CD68^+^ cells were part of Iba-1^+^ cells (Figure S6). Less than 5% of Ly6C^+^ cells were double-positive for CD68 (Figure S6), indicating that Ly6C^+^ cells are mostly a different population than activated microglial cells.

Our results also suggest that Ly6C^+^ cells in the ischemic brains were not purely monocytes/macrophages and included approximately 10% NE^+^ neutrophils and 15% CD3^+^ T cells. However, the infiltrated neutrophils and T cells did not produce BDNF, and only few of them (1–5%) were positive for TNF-*α* or IL-1*β* two days after dMCAO. Among the Ly6C^+^ infiltrated cells, it is probably monocytes/macrophages that are the major contributors for production of BDNF, IL-1*β*, and TNF-*α*.

The spleen was reported to be a major reservoir of undifferentiated Ly6C^+^ cells and proinflammatory cytokines that are readily mobilized during inflammatory processes, including stroke [5]. In this study, we found that dMCAO leads to infiltration of Ly6C^+^ cells into the brain after stroke. It was reported that systemic administration of human umbilical cord blood progenitor cells (HUCBC) significantly reduces the number of macrophage/microglia in the injured brain and maintains the size of the spleen [14]. In our study, the number of infiltrated Ly6C^+^ cells was significantly reduced after MSC infusion. The MSCs that had migrated into the spleen may be responsible for these effects by an immune-inhibitory mechanism [32].

On the other hand, inflammation can also be seen as part of a protective response indispensable for limiting stroke-induced brain damage and inducing repair. The beneficial effects of inflammation at least include direct neuroprotection through neurotrophins.

One of the best understood trophic factors in the context of stroke is BDNF, which promotes neurological recovery following middle cerebral artery occlusion [33]. BDNF can be produced by neurons through neuronal activity-dependent exocytosis. Our previous study showed that BDNF can also be secreted by Iba-1^+^ microglia [7]. Now we have demonstrated that monocytes/macrophages are probably another important participant that mediates the neurotrophic effects after dMCAO. At 48 h after ischemia onset, nearly 32.40% of Ly6C^+^ cells were BDNF-positive. Although MSC infusion reduced the total number of Ly6C^+^ cells in the brain, MSC treatment nevertheless enhanced the total number of BDNF^+^/Ly6C^+^ cells and increased the percentage of BDNF-producing cells among the infiltrated monocytes/macrophages in the infarct area two days after ischemia onset.

In our previous study, we have shown that in the infarct areas, proinflammatory cytokines IL-1*β* and TNF-*α* levels are increased [7]. However, the cell types that have contributed to production of these two cytokines have not been carefully examined. In the current study, we looked mainly into monocytes/macrophages and microglia for cytokine production. Quantification of cells using secretory proteins such as cytokines and growth factors by immunostaining is challenging. Perhaps a better approach to overcome the limitation is quantifying the transcript as validation, which will be employed in our future studies.

In terms of IL-1*β*, 12.30 ± 2.03% of infiltrated Ly6C^+^ cells were positive for IL-1*β* after ischemia, and MSC treatment increased the percentage to 38.62 ± 6.99%. MSC treatment also increased the numbers of IL-1*β* ^+^ and IL-1*β* ^+^/Ly6C^+^ cells from 24.75 ± 7.35 and 12.5 ± 2.18 to 41.00 ± 6.82 and 23.75 ± 3.16 per view field, respectively.

Interestingly, very few Ly6C^+^ cells expressed TNF-*α* before MSC transplantation (0.9 ± 1.0 per view field). After MSC treatment, although the quantity of Ly6C^+^ cells decreased in the brain, the number of TNF-*α* ^+^/Ly6C^+^ cells and its proportion among Ly6C^+^ cells both increased.

Ritzel et al. have shown that, in mice, 72 h after a 90 min MCAO, monocytes/macrophages accumulated in the ischemic brain compared to sham controls. Microglia produce relatively higher levels of reactive oxygen species and TNF-*α*, whereas monocytes/macrophages are the predominant IL-1*β* producer [20]. Unexpectedly, in the current study, Iba-1^+^ microglial cells did not express any significant level of TNF-*α* or IL-1*β*, whether in the infarct area, the intact cortex and striatum, or the corpus callosum. Some CD68^+^ cells (possibly activated microglia in our study) were positive for TNF-*α* or IL-1*β* (Figures 6 and 7). Ischemic damage and MSC transplantation after stroke did not induce the Iba-1^+^ cells to produce TNF-*α* or IL-1*β* either. It seems that, in the current experimental setting and with respect to only microglia and monocytes/macrophages, infiltrated monocytes/macrophages, rather than microglia, are the predominant contributors for IL-1*β* and TNF-*α* production.

In our study, intravenous transplantation of mesenchymal stem cells reduces the number of infiltrated macrophages but enhances the proportions positive for BDNF, TNF-*α*, and IL-1*β* in the infarct cortices of dMCAO rats. The possible mechanisms may include, but may not be limited to, the following.

The majority of infused MSCs are trapped in the lung and spleen. Spleen is one of the organs that play an important role in the periphery after stroke. In the case of severe ischemia, the spleen contracts under the regulation of the hypothalamic-pituitary-adrenal axis and the sympathetic nervous system. This reduction in spleen size is associated with increased release of immune cells and proinflammatory cytokines into the blood, a greater extent of infiltration of leukocytes and monocytes, and a higher level of microglial activation in the brain. It seems that intravenous infusion of MSCs may suppress the “overactivated” inflammation and immune reaction in the spleen, reduce the influx of immune cells, prevent the exhaustion of immune capacities of spleen, and eventually avoid immunosuppression in stroke patients [34].

A noteworthy phenomenon was that only the Ly6C^+^ cells located in the infarct area were positive for BDNF or proinflammatory cytokines IL-1*β* and TNF-*α*. The majority of Ly6C^+^ cells surrounding the infarct area were negative for BDNF, TNF-*α*, and IL-1*β*. Two interesting aspects were implicated in this observation. First, after infusion, MSCs mostly accumulated at the peri-infarct areas too. The spatial correlation suggests that MSCs may have influenced the infiltrated cells through a paracrine and/or cell-cell contact mechanism. Second, Ly6C^+^ cells in the infarct areas could be positive for either BDNF or proinflammatory cytokines (IL-1*β* and TNF-*α*), but not for both, whereas Ly6C^+^ cells at the peri-infarct areas were mostly negative for all three. These results suggest that the infiltrated cells, including monocytes, may be intrinsically heterogeneous and can react differentially to different microenvironments. Microglia can be categorized into M1 and M2 subtypes, and the two subtypes can be switched in a certain context [34, 35]. Microglia originate from the hematopoietic lineage and are derived from the infiltrated monocytes during embryo development [21]. Like microglia, monocytes are possibly heterogeneous as well. Further efforts are warranted to identify the molecular cues in the infarct and peri-infarct areas that can induce infiltrated monocytes to assume different phenotypes. Once identified, these cues may offer a potential candidate for manipulating the functions of macrophages and improve the treatment of stroke.

## Data Availability

The data used to support the findings of this study are available from the corresponding author upon request.

## Supplementary Materials

Supplementary Materials Supplementary Figure 1: Ly6C^+^ cells in ischemic brains are derived from periphery circulation at day 2. Supplementary Figure 2: a small part of Ly6C^+^ cells costained with neutrophil elastase and CD3 at day 2. Supplementary Figure 3: infiltrated neutrophils and T cells minimally contribute to BDNF production at day 2. Supplementary Figure 4: infiltrated neutrophils and T cells minimally contribute to IL-1*β* production at day 2. Supplementary Figure 5: infiltrated neutrophils and T cells minimally contribute to TNF-*α* production at day 2. Supplementary Figure 6: the overlapping of CD68^+^ cells with Ly6C^+^ and Iba-1^+^ cells, respectively, at day 2.
