# Supplementary material for: Intravenous Transplantation of Mesenchymal Stem Cells Reduces the Number of Infiltrated Ly6C+ Cells but Enhances the Proportions Positive for BDNF, TNF-1α, and IL-1β in the Infarct Cortices of dMCAO Rats
Source: Stem Cells Int. 2018 Oct 2;2018:9207678. doi: 10.1155/2018/9207678 (PMC6189688; doi:10.1155/2018/9207678)
Supplement: Supplementary Materials — Supplementary Figure 1: Ly6C+ cells in ischemic brains are derived from periphery circulation at day 2. Supplementary Figure 2: a small part of Ly6C+ cells costained with neutrophil elastase and CD3 at day 2. Supplementary Figure 3: infiltrated neutrophils and T cells minimally contribute to BDNF production at day 2. Supplementary Figure 4: infiltrated neutrophils and T cells minimally contribute to IL-1β production at day 2. Supplementary Figure 5: infiltrated neutrophils and T cells minimally contribute to TNF-α production at day 2. Supplementary Figure 6: the overlapping of CD68+ cells with Ly6C+ and Iba-1+ cells, respectively, at day 2. [file 9207678.f1.docx]

**Supplementary Materials**


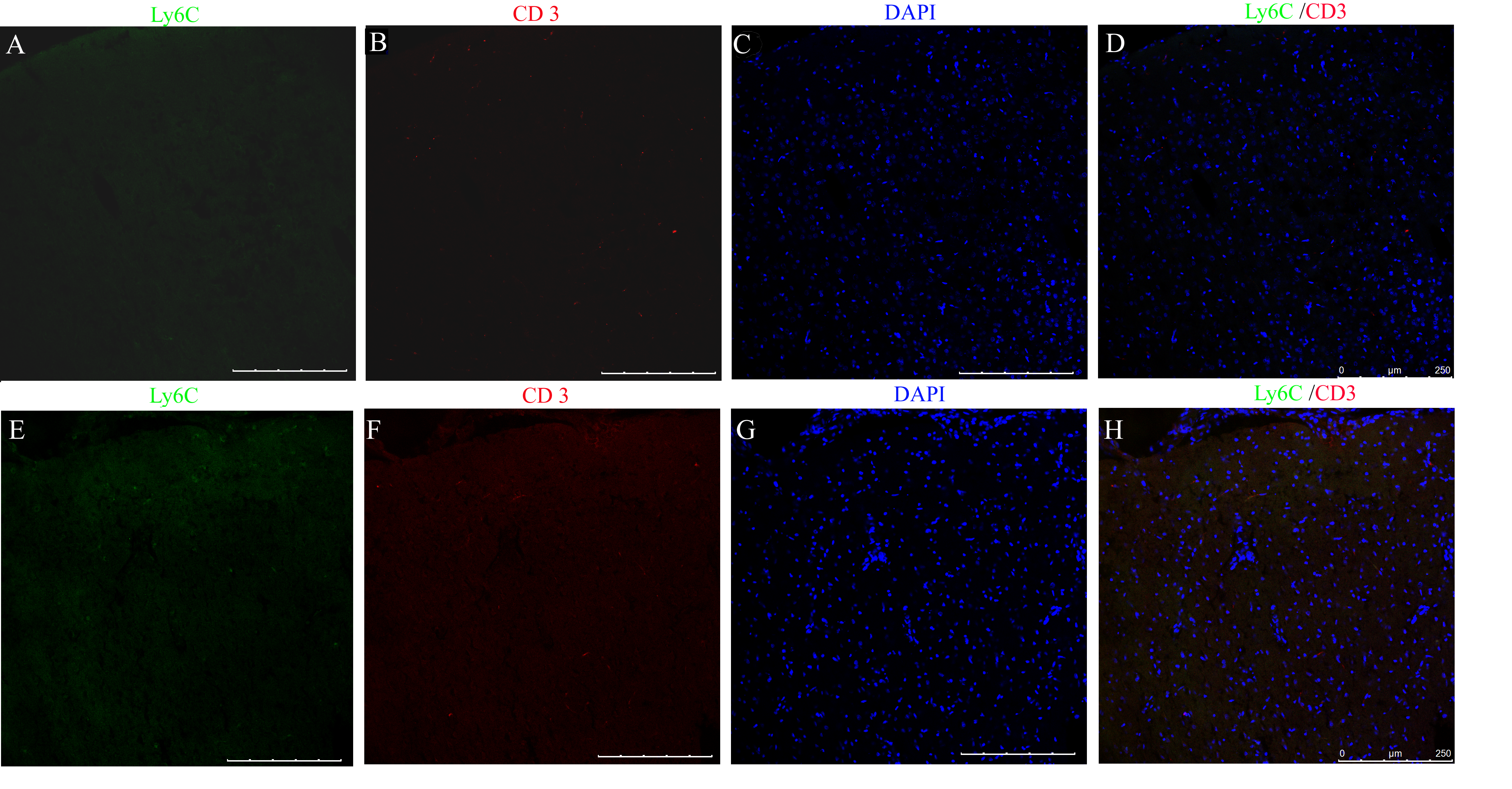


**Supplementary Figure 1. Ly6C+ cells in ischemic brains are derived from periphery circulation at day 2.**

A-D: In the cortex of naïve rats, no Ly6C- or CD3-positive cells were detected. E-H: After depletion of periphery monocytes/macrophages by clodronate liposome treatment, the numbers of Ly6C+ cells and CD3+ cells in the infarct areas were reduced by 80-90%. n=5; scale bar, 250 μm.


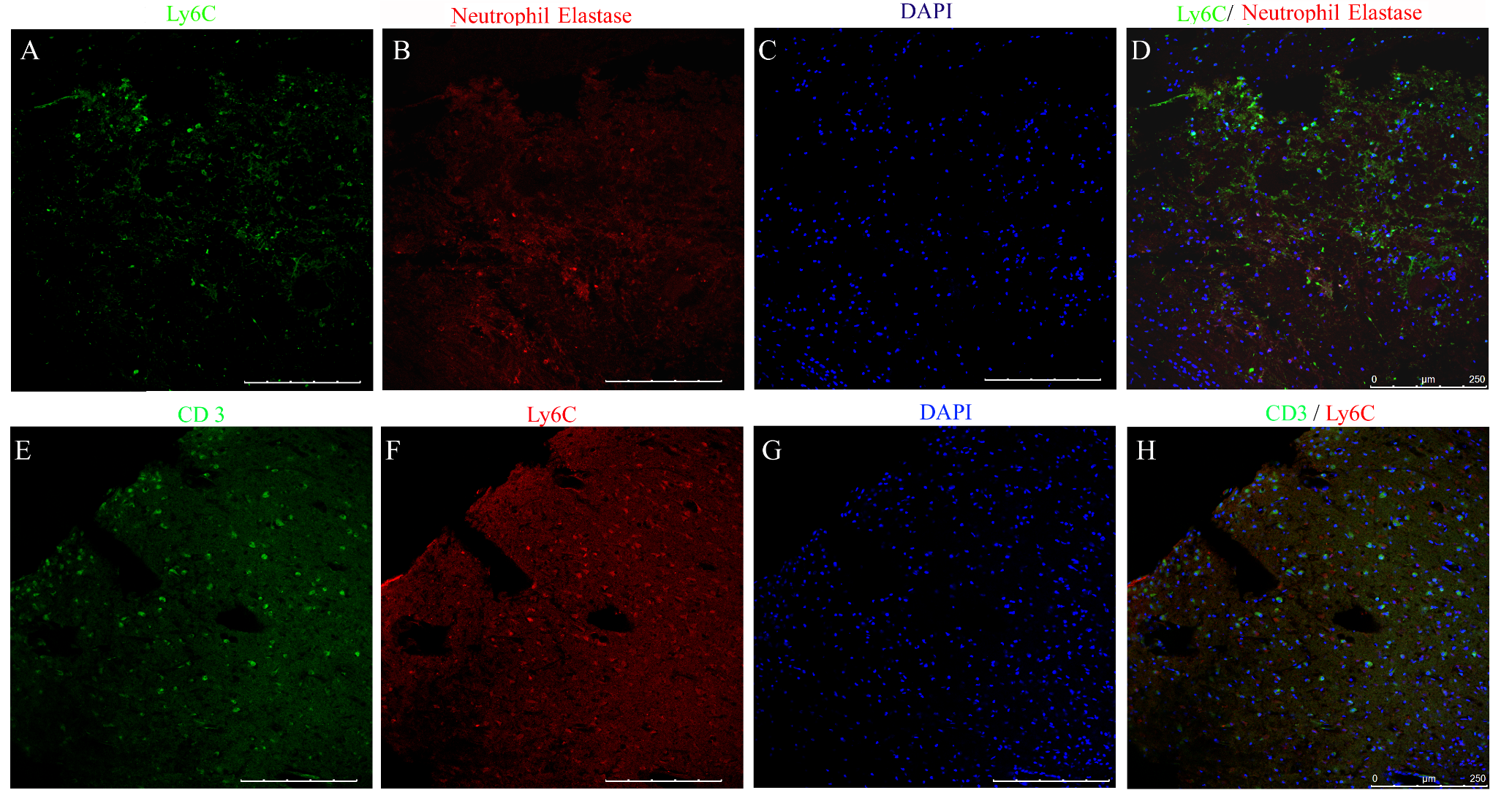


**Supplementary Figure 2. A small part of Ly6C+ cells co-stained with neutrophil elastase and CD3 at day 2.**

A-D: In the cortex of dMCAO rats, about 10% of Ly6C+ cells co-expressed neutrophil elastase (NE). E-H: Around 15% of Ly6C+ cells co-expressed CD3. n=5; scale bar, 250 μm.

**
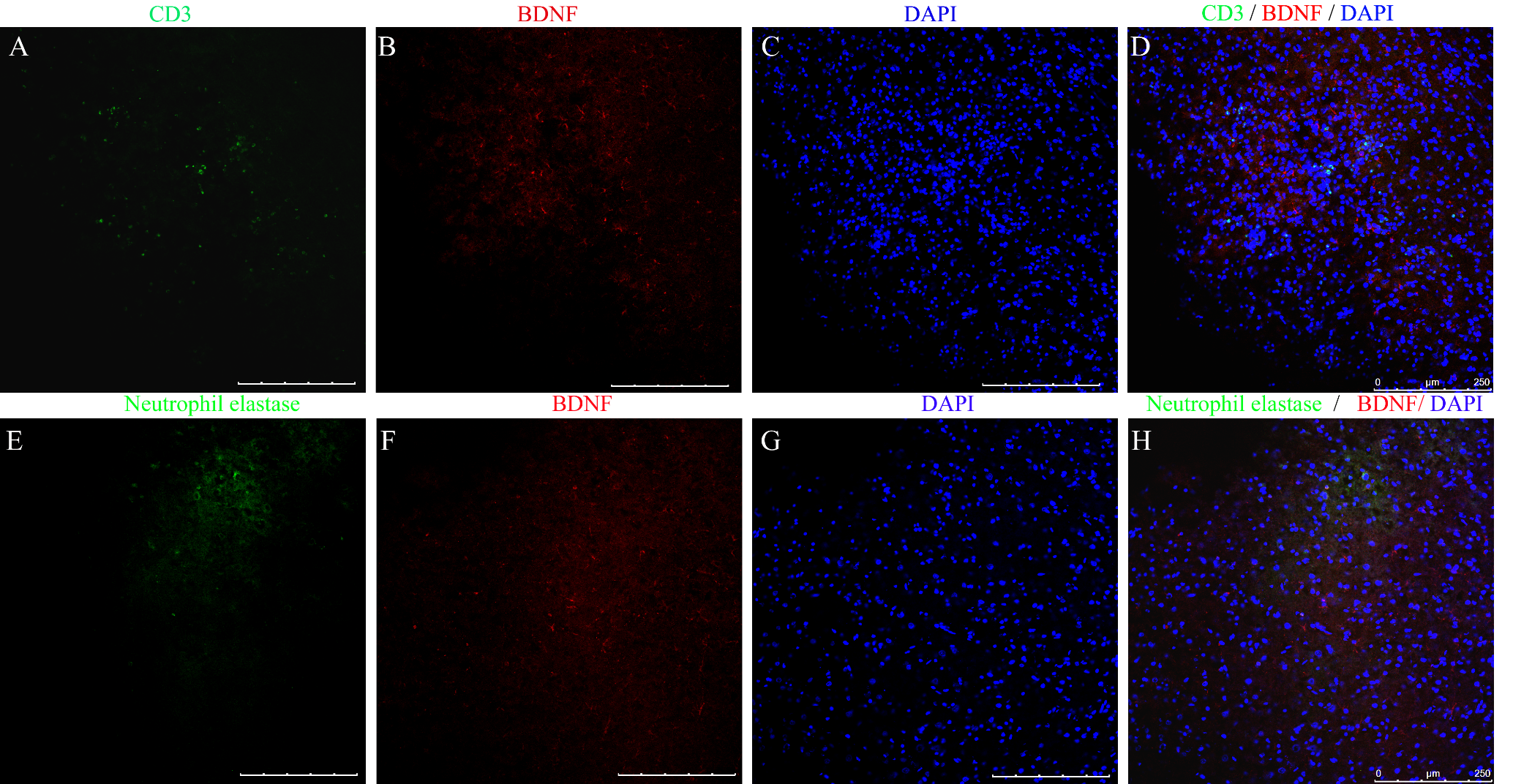
**

**Supplementary Figure 3. Infiltrated neutrophils and T cells minimally contribute to BDNF production at day 2.**

A-D: In the cortex of dMCAO rats, almost no CD3+ cells were positive for BDNF. E-H: less than 1% of neutrophils were positive for BDNF. n=5; scale bar, 250 μm.

**
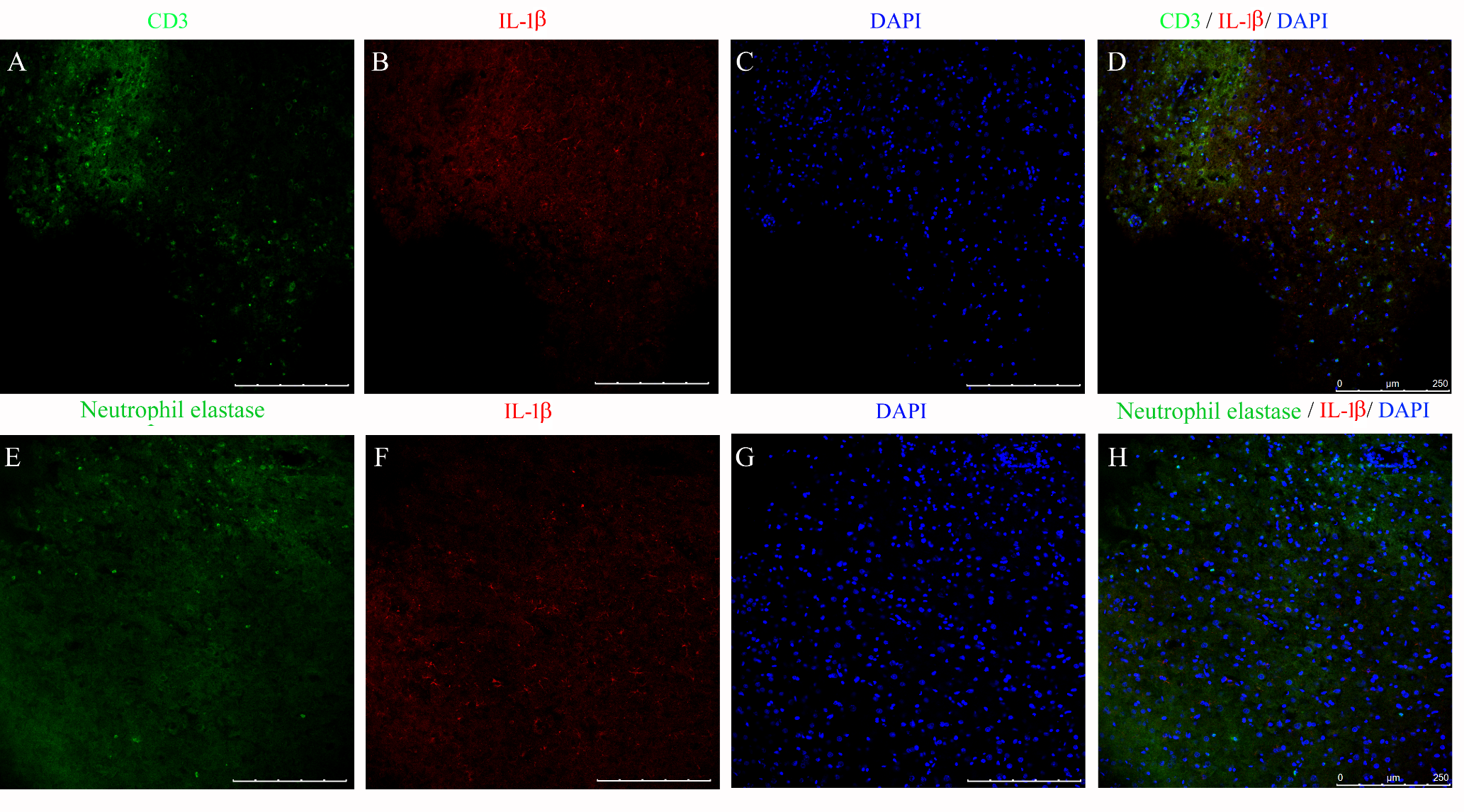
**

**Supplementary Figure 4. Infiltrated neutrophils and T cells minimally contribute to IL-1β production at day 2.**

A-D: In the cortex of dMCAO rats, around 3-5% of CD3+ cells co-expressed IL-1β. E-H: Around 2% of neutrophils co-expressed IL-1β. n=5; scale bar, 250 μm.

**
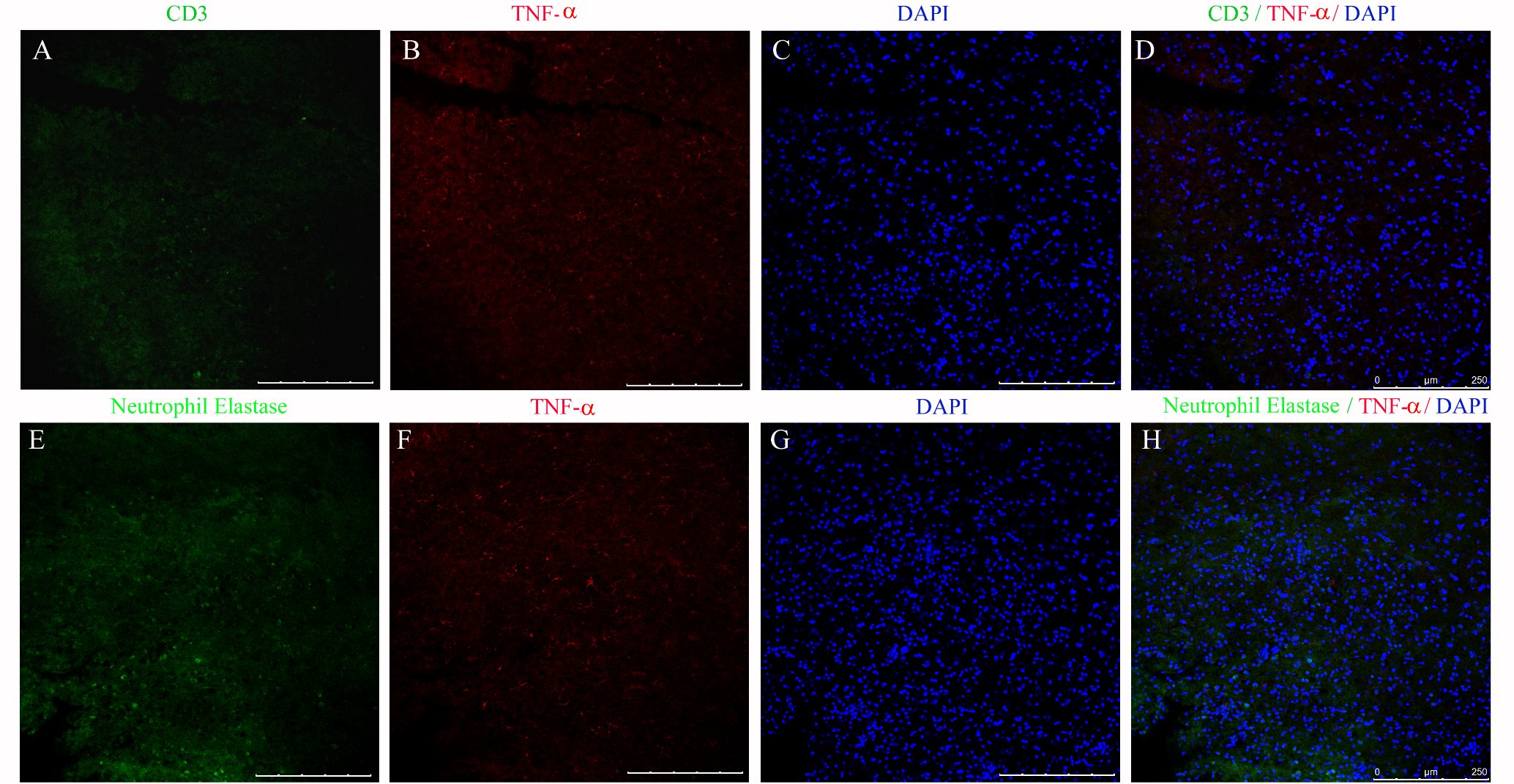
**

**Supplementary Figure 5. Infiltrated neutrophils and T cells minimally contribute to TNF-α production at day 2.**

A-D: In the cortex of dMCAO rats, around 2-4% of CD3+ cells co-expressed TNF-α. E-H: Around 1-2% of neutrophils co-expressed TNF-α. n=5; scale bar, 250 μm.


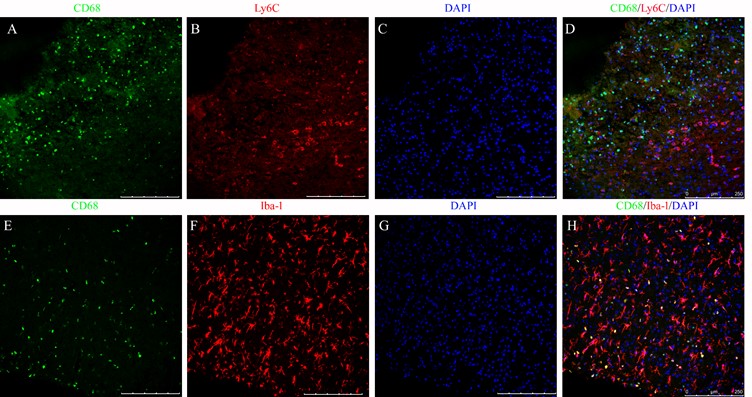


**Supplementary Figure 6. The overlapping of CD68+ cells with Ly6C+ and Iba-1+ cells respectively at day 2.**

A-D: In the cortex of dMCAO rats, 2-5% of Ly6C+ cells were double-positive for CD68. E-H: all of the CD68+ cells were also positive for Iba-1. n=5; scale bar, 250 μm.
